# ALZHEIMER’S DISEASE IN AI/AN COMMUNITIES: THE NEED FOR A CULTURALLY TAILORED ROAD MAP FOR ACTION

**DOI:** 10.1093/geroni/igz038.1336

**Published:** 2019-11-08

**Authors:** William F Benson

**Affiliations:** International Association for Indigenous Aging, Silver Spring, Maryland, United States

## Abstract

Although little is known about Alzheimer’s disease and other dementias among our American Indian and Alaska Native (AIAN) population, the numbers of AIAN elders is growing rapidly as is the overall AIAN population. As noted in The Gerontologist, the number of AIAN elders 65 and older will triple by 2030. Between 2014–2060, the number of AIAN people aged 65 and older living with dementia is projected to grow over 475%. This poses challenges for tribal officials, health/social service providers and families. Among these are limited awareness/knowledge about Alzheimer’s disease, inadequate services and resources, and limited experience in providing proven responses to the needs of those with Alzheimer’s and their families. To help tribes and others serving the AIAN population to respond to such challenges, it was important to develop a menu of options for tribal communities and leaders to consider appropriate to their needs, circumstances and values.

